# Clonal spread of multi-resistant *Gallibacterium anatis* isolates among Iranian broilers and layers

**DOI:** 10.1186/s13567-021-00894-1

**Published:** 2021-02-17

**Authors:** Toloe Allahghadry, Duncan Y. K. Ng, Alireza Dibaei, Anders Miki Bojesen

**Affiliations:** 1 Department of Veterinary and Animal Sciences, Faculty of Health and Medical Sciences, University of Copenhagen, Copenhagen, Denmark; 2 Department of Bacteria, Parasites and Fungi, Statens Serum Institute, Copenhagen, Denmark; 3 Poultry Veterinarian. Private Sector, Qom, Iran

**Keywords:** *Gallibacterium anatis*, Poultry, Genomic diversity, Antimicrobial resistance, *gtxA*

## Abstract

*Gallibacterium anatis* is a common cause of reproductive tract infection in chickens, which leads to reduced egg production and increased mortality. This study was undertaken to investigate prevalence of *G. anatis* in 12 poultry flocks originating from Iranian provinces with leading chicken production and to determine genetic diversity, antimicrobial resistance, and the presence of major antigens of the isolates investigated. Out of the 120 chicken tracheal samples collected and tested, 84 (70%) were positive for *G. anatis*. Genotyping by Pulse Field Gel Electrophoresis and genome sequencing revealed a total of 24 pulsotypes for 71 strains (at a 87% similarity level) and seven genome clusters comprising 21 strains (97% similarity level), respectively. The combination of the two typing methods confirmed the presence of several genotypes originating from a common ancestor affecting poultry yet also suggested that identical clones were shared among chickens within farms and between different farms. The latter finding is to our knowledge the first example of clonal presence of *G. anatis* in epidemiologically unrelated farms. The 21 sequenced strains were characterized against a panel of commonly used antibiotics and showed lowered sensitivity to tetracycline (76.2%) and enrofloxacin (90.5%). The widespread presence of multiresistant *G. anatis* isolates calls for non-antibiotic prophylactics. Three major immunogen genes, *gtxA*, *Gab_1309* and *Gab_2312* were detected in the isolates indicating these antigens likely represent effective vaccine targets. A conserved sequence of the *gtxA* gene across a range of epidemiologically independent strains suggests the use of GtxA for future vaccine development purposes.

## Introduction

The human consumption of poultry meat and eggs has increased rapidly during the past century in Europe and the USA, and particularly in developing countries [1]. In Iran, poultry production is among the most important economic activities and with a current production of over 2 million metric tons of chickens a year, it is among the 10 biggest producers of chickens in the world [2]. Three types of chicken enterprises including broiler and layer production, and raising of replacement pullets predominate. Based on the Iranian Veterinary Organization report from 2019, there are about 449 million broilers and 107 million battery-cage layers in the country.

*Gallibacterium anatis* is distributed globally as an important opportunistic pathogen in different poultry production systems [3]. The bacterium has been reported in several countries within Europe (Switzerland, Denmark, Germany, Norway, England, Sweden, Czech Republic, Austria), Africa (Nigeria, Egypt, Morocco), Asia (China, Taiwan, Iran, Syria, India and Japan) and American countries (USA, Canada, Colombia, Peru and Mexico) [3]. *G. anatis* is a Gram-negative, non-motile, encapsulated coccobacillus that constitutes part of the normal microbiota in the upper respiratory tracts and the lower genital tracts of healthy chickens [4]. However *G. anatis* has also been associated with a wide range of lesions and reproductive tract disorders including salpingitis and peritonitis, and is thus considered a highly important opportunistic pathogen that can lead to lowered egg production and increased mortality [5]. The mortality rate can be influenced by several factors such as poor hygiene, inadequate biosecurity measures and concurrent diseases with other poultry pathogens such as *Escherichia coli* [6]. There is a growing concern about the emergence and spread of multidrug resistant strains of *G. anatis*, which have shown lowered sensitivity to a range of antimicrobials including sulpha drugs, novobiocin, tylosin, clindamycin, tetracycline and penicillin [7, 8]. Tetracycline resistance genes have previously been reported to be very common in a larger collection of *G. anatis* field strains from Mexico and Denmark [9]. To counteract the negative effects of antimicrobially resistant *G. anatis* strains, efficient prophylactic measures appear to be the logical alternative to antibiotics to prevent and control *G. anatis* infections in poultry production systems. A pan-genomic reverse vaccinology (RV) approach has previously been applied to identify novel and potentially broadly protective immunogens from *G. anatis* that resulted in a selection of five proteins GtxA, FlfA, Gab_2156, Gab_1309 and Gab_2312 [10]. Based on previous reports, particularly the RTX toxin, GtxA, has the potential to induce protective immunity against *G. anatis* challenge [11]. GtxA is responsible for the hemolytic and leukotoxic properties of *G. anatis* and is considered the most important virulence factor of *G. anatis* [12]*.* GtxA is composed of two domains, a C-terminal is responsible for hemolytic function and a N-terminal domain required for complete hemolytic activity [13]. To allow colonization, *G. anatis* adheres to epithelial cells in avian hosts. The predominant adhesin belongs to the F17-like fimbrial family, which promotes adherence to host mucosal surfaces through binding to *N*-acetyl-d-glucosamine receptors [14]. The fimbrial protein (FlfA) is very important for virulence in vivo and it was demonstrated that a *flfA* knockout mutant (∆*flfA*) is considerably attenuated in chickens [15]. The remaining potential immunogens are less well characterized. Gab_1309 is a membrane protein related to metalloendopeptidases; Gab_2312 is an autotransporter adhesin and Gab_2156 is a F17-like fimbrial subunit [16].

The aim of the present study was to determine the prevalence of the Iranian *G. anatis* strains and to evaluate their genetic diversity, antibiotic susceptibility profiles and content of potential antigens from two major poultry production systems (Battery-cage layer and Broiler) located in the leading provinces of chicken productions in Iran (Tehran, Sari, Qom). Twelve flocks originating from two levels of biosecurity and three geographical locations were included.

## Materials and methods

### Isolation and identification of *G. anatis* from different chicken production systems in Iran

The chicken flocks investigated were selected to represent different production systems and climatic zones in Iran. A stratified random selection was attempted from database registration lists. However, farmers were not always willing to participate, which is why selection had to be made at convenience in some instances. None of the participants, at any level, had any prior knowledge of the presence of *Gallibacterium* infections during the investigation. A total of twelve chicken flocks, comprising battery-cage layers and broilers in three regions (Tehran, Qom, Sari) in Iran were included. The three provinces are central to the Iranian poultry production and can be considered to represent the main production types (broiler and layers) in Iran [2].

Twenty samples were collected from the Tehran province on the north of the central plateau of Iran. Fifty samples originated from the Sari province on the southern coast of the Caspian Sea and fifty samples were collected from the Qom province on the boundary of central Iran. Ten chickens from each flock were swabbed in the trachea using a dry, sterile cotton swab which was immediately smeared onto a blood agar plate. The biosecurity level of each production system was characterized based on the information from the farmers and our own observations during sampling. The classification of the biosecurity levels largely adhered to previously published definitions [4] and Terrestrial Animal Health Code guidelines on biosecurity procedures in poultry production issued by the World Organization for Animal Health in 2019 [17]. The difference in prevalence of infected chickens with *G. anatis* between different biosecurity levels was tested using the Exact Poisson Method [18]. The biosecurity level and the provinces where the farms were placed are recorded in Table 1.

**Table 1:** ***G. anatis*** **isolates included in the study and their origin**

Chickens sampled from flock	Biosecurity level	*G. anatis* positive culture	Province
Battery-cage layer	High	2	Tehran
Battery-cage layer	Moderate	10	Tehran
Battery-cage layer	Moderate	6	Qom
Battery-cage layer	High	3	Qom
Battery-cage layer	Moderate	9	Qom
Battery-cage layer	Moderate	8	Sari
Battery-cage layer	Moderate	8	Sari
Battery-cage layer	Moderate	8	Sari
Broiler	Moderate	8	Sari
Broiler	Moderate	7	Sari
Broiler	Moderate	8	Qom
Broiler	Moderate	7	Qom

Twelve farms were selected for samples collection. A total of 120 chickens’ samples (10 per flock) were collected during the period from June to September 2016.

### Culture-based identification

Tracheal swabs were smeared on blood agar base [Oxoid], supplemented with 5% citrated bovine blood and incubated overnight at 37 °C. Suspect *Gallibacterium* colonies were subcultured on blood agar to obtain pure cultures. *G. anatis* isolates were identified by a wide beta hemolytic zone (1 to 2 mm), smooth and shiny, greyish, semi-transparent, circular as well as raised colonies with an entire margin and a butyrous consistency [19]. Frozen stock were subsequently made from overnight incubated cultures in brain heart infusion broth [BHI] [Difco] at 37 °C, 700 µL of BHI was mixed with 300 µL sterile glycerol 50% and stored at −80 °C until further use.

### Polymerase chain reaction (PCR)

The primers and the probe were predicted to be specific for *G. anatis* and not able to amplify DNA of other members of *Pasteurellaceae* under the PCR conditions chosen [20]. *G. anatis* strain 12656-12, originally isolated from a chicken in Denmark was included in the PCR as a positive control. Briefly, 35 cycles with an initial cycle at 95 °C for 5 min, denaturation at 95 °C for 30 s, annealing at 55 °C for 30 s, extension at 70 °C for 30 s and a final extension at 70 °C for 10 min were run. The PCR products were visualized by agarose gel (1%) electrophoresis and stained with ethidium bromide.

### Matrix assisted laser desorption ionization-time of flight (MALDI-TOF)

MALDI-TOF-based identification was performed on the freshly purified *G. anatis*-suspect colonies according to procedures previously described [21]. The isolates were identified to the species level by MALDI-TOF (Vitek MS RUO; bioMérieux, France) using *E. coli* ATCC 8739 as reference strain and Saramis™ 3.5 (bioMérieux) for spectra interpretation at the Department of Veterinary and Animal Sciences, section of Veterinary Clinical Microbiology, University of Copenhagen, Denmark.

### Genome analysis of *G. anatis* from chicken production systems in Iran

#### Pulse field gel electrophoresis (PFGE)

To genotype the Iranian *G. anatis* strains, all but 13 strains, which were lost due to freezer breakdown, were characterized by PFGE. The typing method was performed according to the standard PFGE protocol for *G. anatis* using ApaI enzyme of choice to evaluate genetic diversity of the strains and identification of clonal lineages among the isolates investigated [22]. The DNA banding patterns were analyzed with Gelcompar II software, the strain similarity was calculated by Dice coefficient to define pulsotypes with position tolerance and optimization set at 1.0% and 2.0%, respectively. The unweighted pair group method with arithmetic mean (UPGMA) was used for cluster analysis [23]. The similarity threshold obtained in this study was based on three band differences among the pulsotypes, as a banding pattern difference of one to three bands in PFGE indicates that the isolates are closely related [24].

#### Whole genome sequencing (WGS)

A total of 21 strains representing all twelve farms sampled were selected for whole genome sequencing based on the PFGE typing data with the aim of including as much diversity as possible. The strains sequenced in the current study represented both major production systems originating from the three central provinces in poultry production in Iran. The Maxwell RSC cultured cell DNA kit was used to purify DNA from the *G. anatis* strains according to the manufacturer’s instruction (Maxwell ® RSC Cultured Cells DNA Kit). The DNA concentration and quality were measured using a NanoDrop spectrophotometer. Whole genome sequencing was performed using Illumina Nextera XT and MiSeq reagent kit v3 at the Department of Veterinary and Animal Sciences, section of Veterinary Clinical Microbiology, University of Copenhagen, Denmark. The *G. anatis* UMN179 originally isolated in Minnesota, USA, was used as the reference (CP002667). The reads were assembled using SKESA 2.3.0 [25], annotated with Prokka 1.14.0 [26] and analyzed with Roary 3.12.0 [27]. Single nucleotide polymorphisms (SNP) were called and filtered using Snippy 4.4.5 [28] and a maximum likelihood phylogenetic tree was calculated in IQ-TREE 1.6.12 using default setting, 100 bootstrap replications were included [29]. The tree was rooted with *G. anatis* UMN179 to characterize the pan-genome of *G. anatis*. Abricate 0.9.3 was used to detect virulence genes and antibiotic resistant genes (Resfinder) [30].

### *gtxA* gene comparison

We undertook a comparison of *gtxA* toxin gene from the Iranian isolates and 27 *G. anatis* strains from different parts of the world (Denmark, USA, Germany, Mexico, Czech Republic) (Table 2). CLC Genomics workbench 7.4 was used to analyze and visualize the sequencing data. The aligned sequences were used to assess the *gtxA* sequence conservation and to find possible evolutionary relationship among all of the strains using Clustal Omega and maximum likelihood, respectively [31].

**Table 2:** ***G. anatis*** **strains from all over the world as the reference strains for comparison of** ***gtxA*** **toxin gene with the Iranian isolates**

Strain	Country	Host	Isolation source
UMN179	USA	*Gallus gallus*	Peritonitis
CCM 5976	Czech Republic	*Gallus gallus*	Oviduct
CCM 5974	Czech Republic	*Gallus gallus*	Liver
CCM 5995	Czech Republic	*Gallus gallus*	ND
4895	Mexico	*Gallus gallus*	ND
12158	Denmark	*Gallus gallus*	Salpingitis
23K10	Denmark	*Gallus gallus*	Cloacae
21K2	Denmark	*Gallus gallus*	Cloacae
F149	Denmark	*Anas Platyrhynchos*	Intestine
7990	Mexico	*Gallus gallus*	ND
IPDH697-78	Germany	*Gallus gallus*	ND
10672-6	Denmark	*Gallus gallus*	Salpingitis
10672/9	Denmark	*Gallus gallus*	ND
23T10	Denmark	*Gallus gallus*	Trachea
Avicor	Mexico	*Gallus gallus*	Heart
1797	Mexico	*Gallus gallus*	Joint
F0003406	USA	*Meleagris gallopavo*	Liver
Gerl 4224-88	Germany	*Aves*	ND
36961/SV7	Denmark	*Gallus gallus*	Trachea
21T2	Denmark	*Gallus gallus*	Trachea
20558/3K1	Denmark	*Anser*	Cloacae
CCM 5975	Czech Republic	*Gallus gallus*	ND
Gerl 2740/89	Germany	*Columba*	ND
F279	Denmark	*Anas Platyrhynchos*	Intestine
18102/2	Denmark	*Anas Platyrhynchos*	Brain
Gerl 3348/80	Germany	*Columba*	ND
12656-12	Denmark	*Gallus gallus*	Liver

### Antimicrobial susceptibility testing

The broth microdilution method was performed to determine the antimicrobial sensitivity of the 21 sequenced *G. anatis* isolates against eleven antimicrobials using a commercially prepared Sensititre Veterinary MIC plate following the suggested manufacturer protocol (Thermo Scientific Sensititre Veterinary MIC Plates, USA). The antimicrobial substances and concentration ranges are recorded in Table 3. Determination of minimum inhibitory concentration (MIC) was performed according to the CLSI standard VET08, M31-A2 and M100 [32–34]. Results were read using the Thermo Scientific™ Sensititre™ SWIN™ Software System.

**Table 3:** **Antimicrobial resistance of the 21 sequenced Iranian** ***G. anatis*** **isolates**

Antimicrobial	Concentration (μg/mL)	No. of isolates (%)		
		Susceptible	Intermediate	Resistant
Amikacin	4–32	95.2	0.0	4.8
Amoxicillin/clavulanic acid	0.25/0.12 to 8/4	66	34	0.0
Cefazolin	1–32	23.8	71.4	4.7
Ceftazidime	4–16	80.9	9.5	9.5
Chloramphenicol	2–32	95.2	4.8	0.0
Doxycycline	0.25–8	81	14.3	4.7
Enrofloxacin	0.125–4	0.0	9.5	90.5
Gentamicin	0.25–8	76.2	9.5	14.3
Imipenem	1–8	76.2	9.5	14.3
Tetracycline^a^	4–16	23.8	0.0	76.2
Trimethoprim/sulfamethoxazole	0.5/9.5–4/76	90.5	0.0	9.5

^a^Out of the 21 strains tested, the identical outcomes were confirmed in phenotypic-genotypic evaluation of resistance to tetracycline.

## Results

### Isolation and identification of *G. anatis* from chicken production systems in Iran

#### Culture-based identification

Among the 120 samples tested by culture-based identification, 84 samples showed positive growth of bacteria with a *G. anatis*-suspect colony morphology and strong beta-hemolysis. A total of 30 out of 40 (75%) broilers and 54 out of 80 (68%) battery-cage layers sampled positive for *G. anatis*.

The Exact Poisson Method was used to compare the incidence rate (0.79) of *G. anatis* on moderate level biosecurity farms to the incidence rate (0.25) on farms with a high level of biosecurity, respectively. The incidence rate ratio of 3.16 was highly different at a 95% level of significance (*p* = 0.0084) indicating that the prevalence proportion of *G. anatis* in farms with moderate level of biosecurity is higher than farms with a high level of biosecurity.

#### Polymerase chain reaction (PCR)

The purified colonies were identified by *Gallibacterium* PCR. Among all the 84 positive samples identified by culture-based identification, a fragment of 120 bp was produced from the PCR method identical to the positive control *G. anatis* 12656-12.

#### Matrix assisted laser desorption ionization-time of flight (MALDI-TOF)

All of the 84 purified colonies were identified at the species level as *Gallibacterium anatis* by MALDI-TOF, using *E. coli* (ATCC 8739) as reference strain and Saramis™ 3.5 (bioMérieux) for spectra interpretation.

### Genome analysis of *G. anatis* from chicken production systems in Iran

#### Pulse field gel electrophoresis (PFGE)

**Figure 1:**
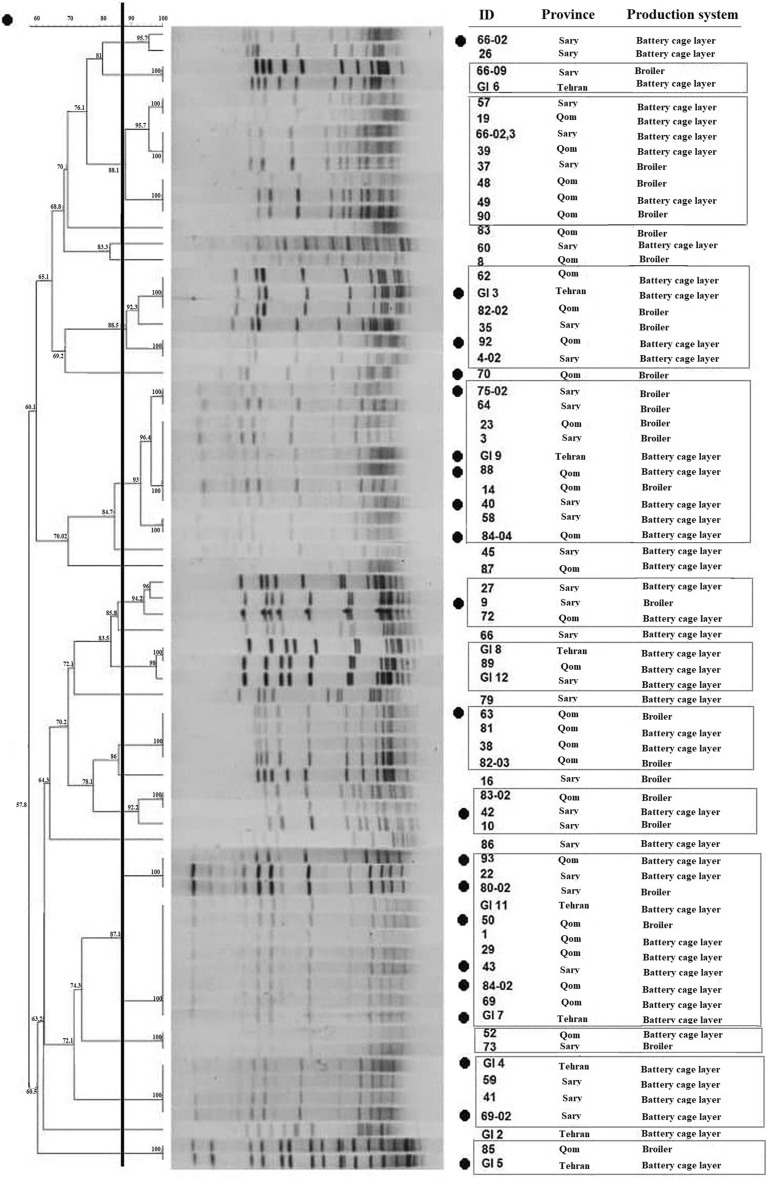
**ApaI dendrogram of 24 clusters (similarity ≥ 87% vertical line) of 71** ***G. anatis*** **isolates, derived from the Dice coefficient and UPGMA using Gelcompar II software.** A PFGE cluster was defined as a group of isolates with a similarity of ≥ 87% of their Dice coefficients with position tolerance and optimization set at 1.0% and 2.0%, respectively. The samples are from different sources (Broiler and Battery-cage layer) and different origins (Tehran, Qom, Sari) in Iran. Identical clones among 71 *G. anatis* isolates from different origins and different production systems were grouped individually. The 21 representative strains for WGS are labelled accordingly (black circle).

Chromosomal DNA fingerprinting of the 71 *Gallibacterium* isolates was carried out by comparing the ApaI digestion patterns obtained after standard PFGE method for *G. anatis*, (13 strains were lost during storage at −80 °C due to a freezer breakdown). The PFGE banding patterns consisted of 12 to 17 DNA fragments sized between 20.5 and 1135 kb. Identical PFGE groups were indicated by a threshold at an 87% similarity of the Dice coefficient, which corresponded to a maximum of three band differences among the profiles. A total of 24 clusters were identified at an 87% similarity level (Figure 1). Strains having identical PFGE patterns are defined as being a single clone. The results demonstrate that more than a single clone was apparent in each flock indicating the presence of multiple *G. anatis* clonal lineages in the Iranian poultry. Both identical and different *G. anatis* clones were found among the isolates from different Iranian production systems and farms, respectively. In fact, our results indicate a common presence of identical strains at different farms keeping broilers and battery-cage layers. In example, *G. anatis* strains from the Tehran province had identical PFGE patterns to those of isolates from different farms from the Sari and Qom provinces, respectively. The identical clones of *G. anatis* between different origins investigated are shown in Figure 1.

### Whole genome sequence analysis of Iranian *Gallibacterium anatis* strains

**Figure 2:**
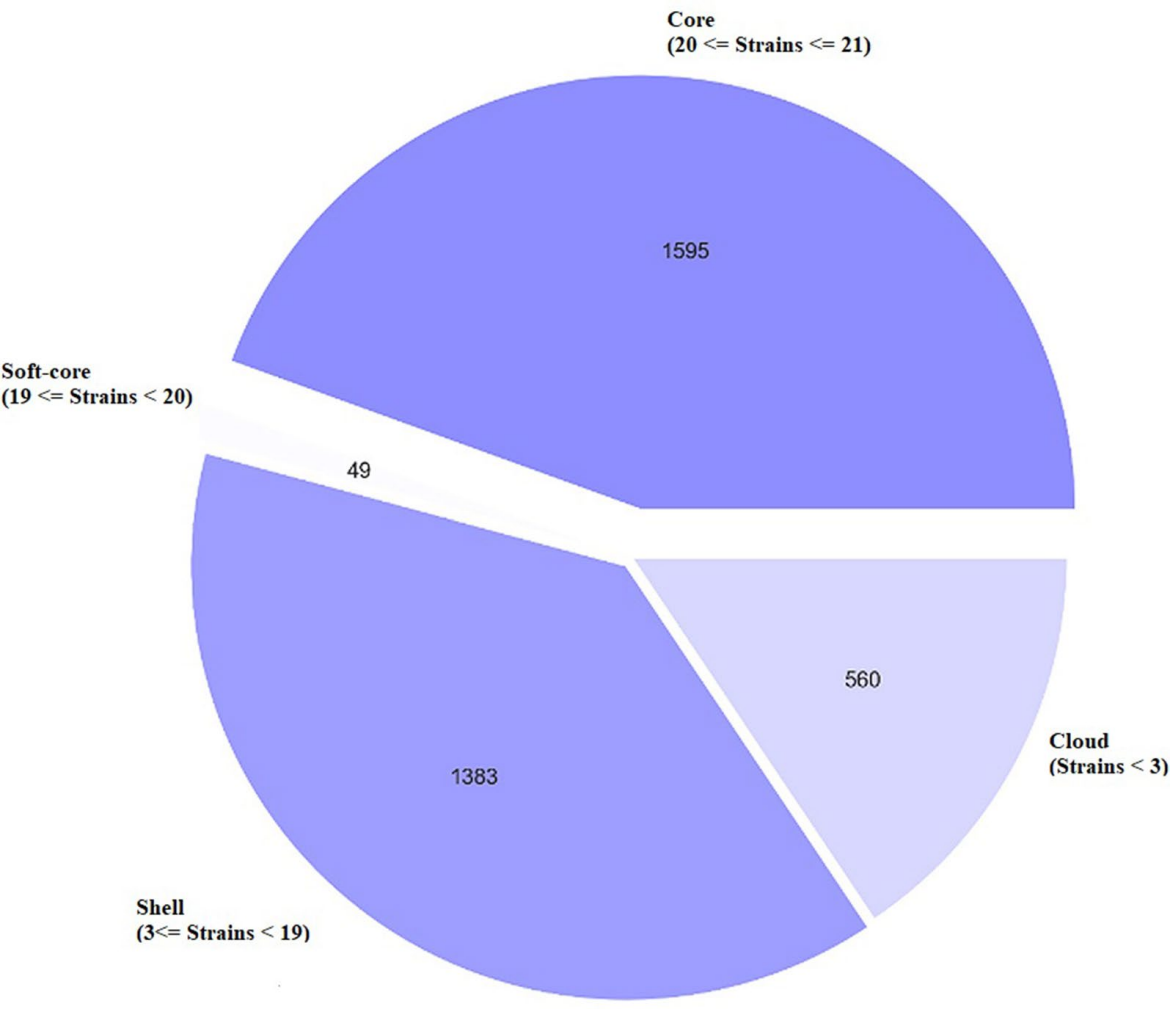
**Pan-genome analysis of the 21 Iranian isolates of** ***G. anatis*.** Pie chart, showing the distribution of the different type of genes and the number of isolates presented in *G. anatis* with the use of Roary software. The Pie chart composed of core (44.4%), soft-core (1.3%), shell (38.5%) and cloud (15.6%) genes.

**Figure 3:**
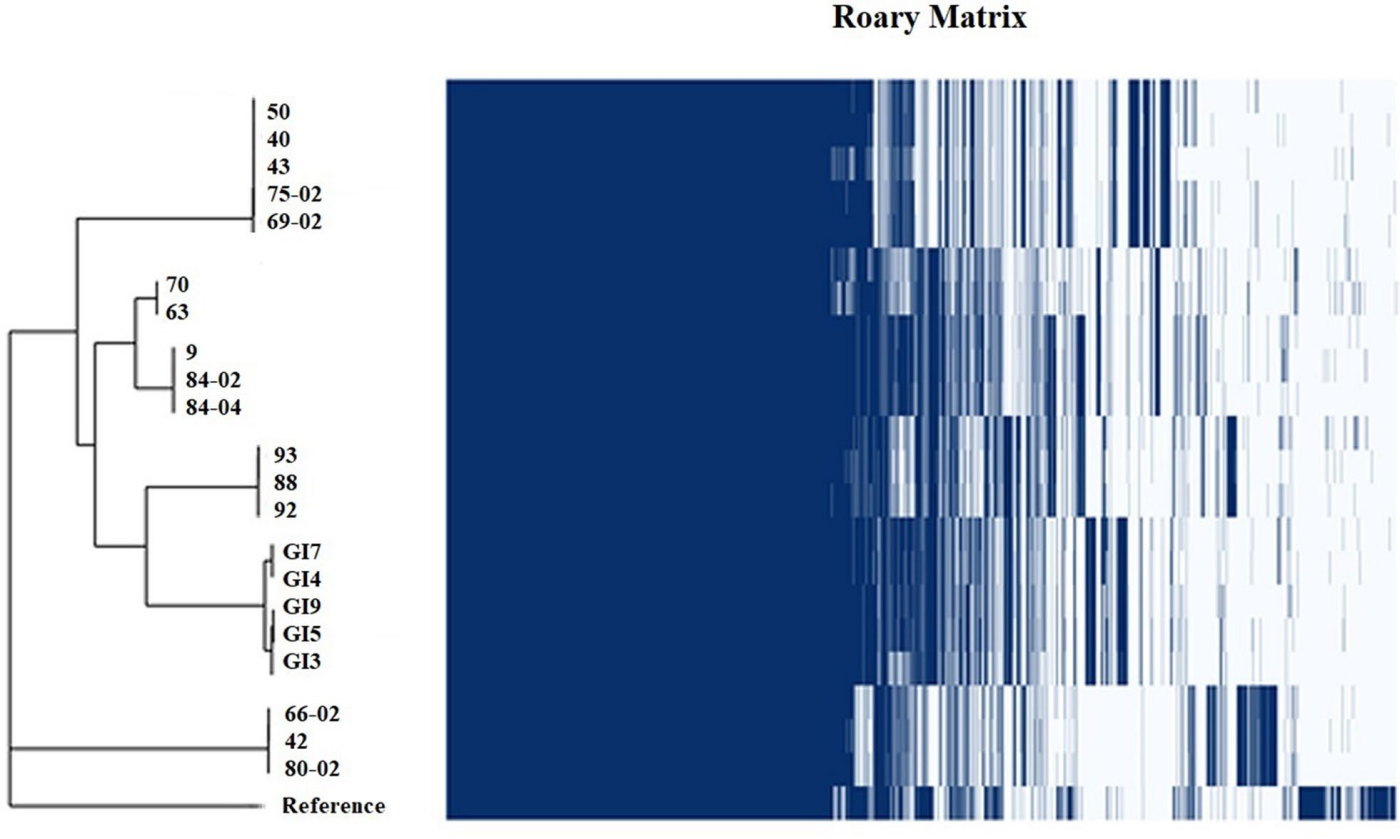
**Pan-genome analysis of the 21 Iranian isolates of** ***G. anatis*.** Presence and absence matrix against the core SNP phylogeny. Evolutionary insights between the isolates based on the core genome (left panel-sequence similarity > 97%). The core genome tree generated was compared with a matrix where the core and accessory genes were either present or absent (right panel). Dark blue: gene present across the strains.

**Figure 4:**
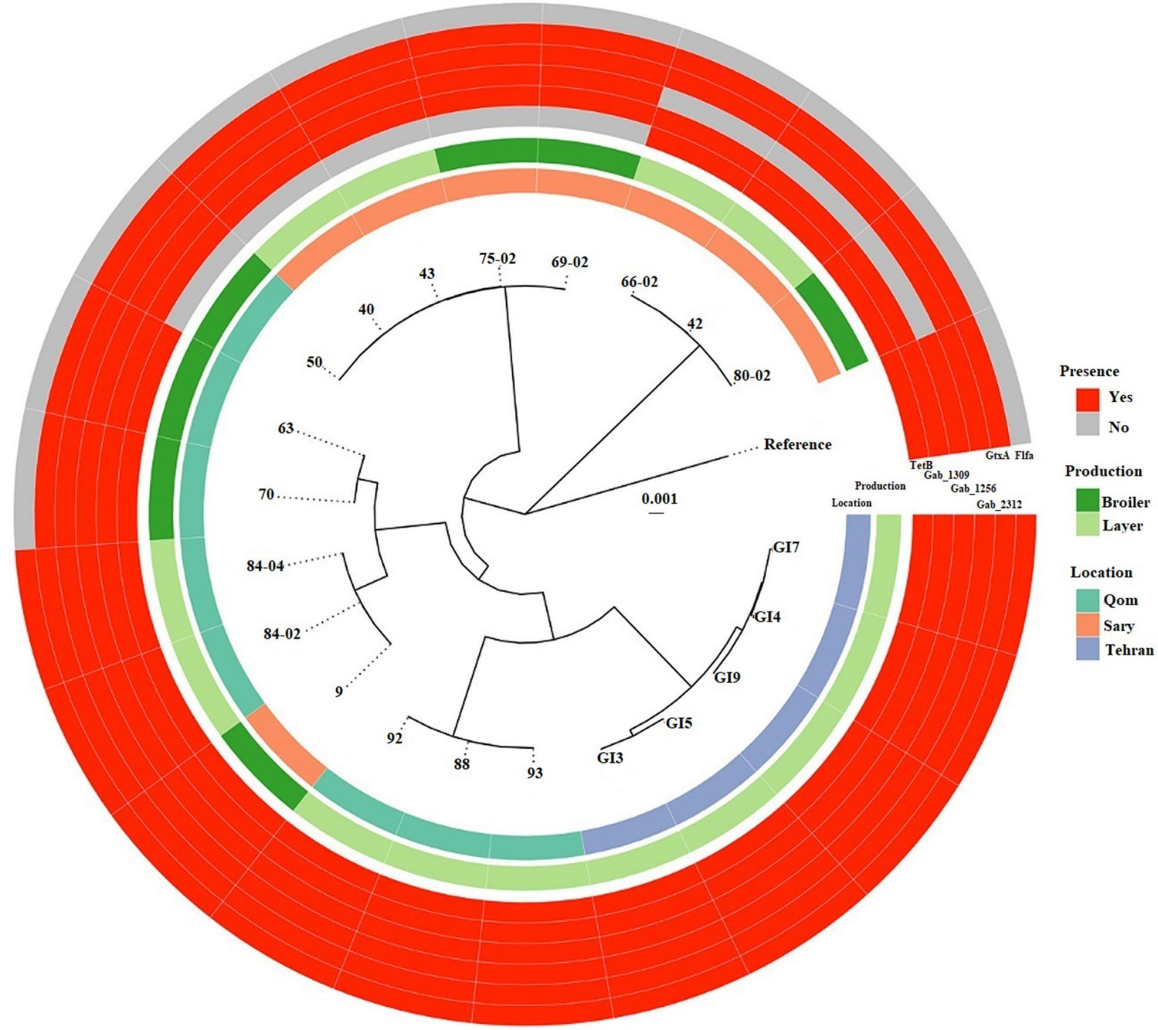
**Phylogenetic tree of the 21 Iranian isolates of** ***G. anatis*** **and the reference strain (*G. anatis*** **UMN179).** The scale bar represents substitutions per site. The sample locations, production system, immunogenic proteins (GtxA, FlfA, Gab_1309, Gab_2312, Gab_2156) and antimicrobial resistance of *G. anatis* are examined in this study. Location and production system are labelled accordingly. Immunogenic proteins data are classified as present in red and absent in grey based on blast results. TetB represents tetracycline resistance gene *tetB*.

The 21 strains of *G. anatis* were subjected to Illumina Nextera XT and MiSeq reagent kit v3 sequencing. The pan-genome of 21 *Gallibacterium* strains was estimated to consist of 3886 genes. The strains grouped into seven clusters. Roary was used to determine which genes were the most prevalent in the 21 strains and to categorize them accordingly. The core genome consisting of genes present in all genomes and the soft-core representing genes present in 95% of the genomes, respectively, both represent a pool of highly conserved genes. The shell and cloud genomes contain accessory genes present occasionally or in single strains, respectively. Based on the pie chart which essentially is a breakdown of the abundance, the results summarized 1595 genes present in all 21 isolates (Core genes), 49 genes in 19 isolates (Soft-core genes), 1383 genes in 3–19 isolates (Shell genes) and 560 genes in less than 3 isolates (Cloud genes) (Figure 2). Based on the results extracted from pan-genome analysis, the sequence similarity in each cluster was significantly higher at the core genome level while the strains in each cluster could differ more at the accessory genome level. As indicated in the frequency plot of the Roary matrix, all the clades had unique genes associated with them (Figure 3). Based on the pan-genome analysis, it was demonstrated that the core genome made up 46% of the whole genome leaving 54% to the accessory genome. The core genome-based single nucleotide polymorphism (SNP) was assessed to provide an accurate evolutionary estimate among the strains. The tree was analyzed using the maximum likelihood method based on 69 735 SNPs (Figure 4). *G. anatis* UMN179 was used as the reference strain (CP002667). Regarding the analysis of the phylogenic tree, the isolates of GI3, GI4, GI5, GI7, GI9, 88, 92 and 93 appeared to be monophyletic and more closely related to strains 9, 84-02, 84-04, 70, and 63 than to strains 50, 40, 43, 69-02 and 75-02. The phylogenic tree comprised six clades plus the two monophyletic strains 66-02, 42 and 80-02. Strains GI3, GI4, GI5, GI7 and GI9, from the Tehran province, clustered at a core genome level with sequences separated by less than 19 to 1592 SNPs rendering these strains more than 99.9% similar. Strains 9, 84-02, 84-04, 70, and 63 of different origins belonged to two separate lineages, one including strains 9, 84-02 and 84-04 separated by only 2–7 SNPs, and another lineage based on strains 63 and 70 separated from the first lineage by 5736 and 5780 SNPs, respectively. The strains 50, 40, 43, 69-02 and 75-02 of mixed origin clustered together and were almost identical with sequences separated by 1 to 149 SNPs. The three strains, 42, 66-02, and 80-02, made up yet another cluster of highly similar strains with sequences separated by 15–39 SNPs, respectively. The sequence of the reference strain (UMN179) from the USA separated from the 21 Iranian strains by 28 744 to 35 898 SNPs. Analysis of the dataset represented more than 97% sequence similarity among the strains at the core genome level.

The distribution of the immunogen encoding genes *gtxA*, *flfA*, *gab_1309*, *gab_2312* and *gab_2156*, respectively, was identified and grouped using the phylogenetic approach described above. The *gtxA*, *gab_1309* and *gab_2312* genes were detected in all 21 Iranian *G. anatis* strains whereas the two virulence genes *flfA* and *gab_2156* were identified in 11 and 18 strains, respectively. *gab_2156* gene was identified in all strains except strains 66-02, 42 and 80-02, all from Sari*.* All 21 sequences were submitted to ResFinder with the aim to identify antibiotic resistance genes. Sixteen strains, excluding strains 40, 43, 50, 69-02 and 75-02, were positive for the tetracycline resistance gene *tetB*, which was located together with *tetC* and *tetR* on a Tn10 transposon. Out of 21 strains investigated, the *ybhf-1* gene was detected in five strains encoding a protein as part of a multidrug ABC transporter system involved in antimicrobial efflux system.

### The *gtxA* toxin gene sequencing

**Figure 5:**
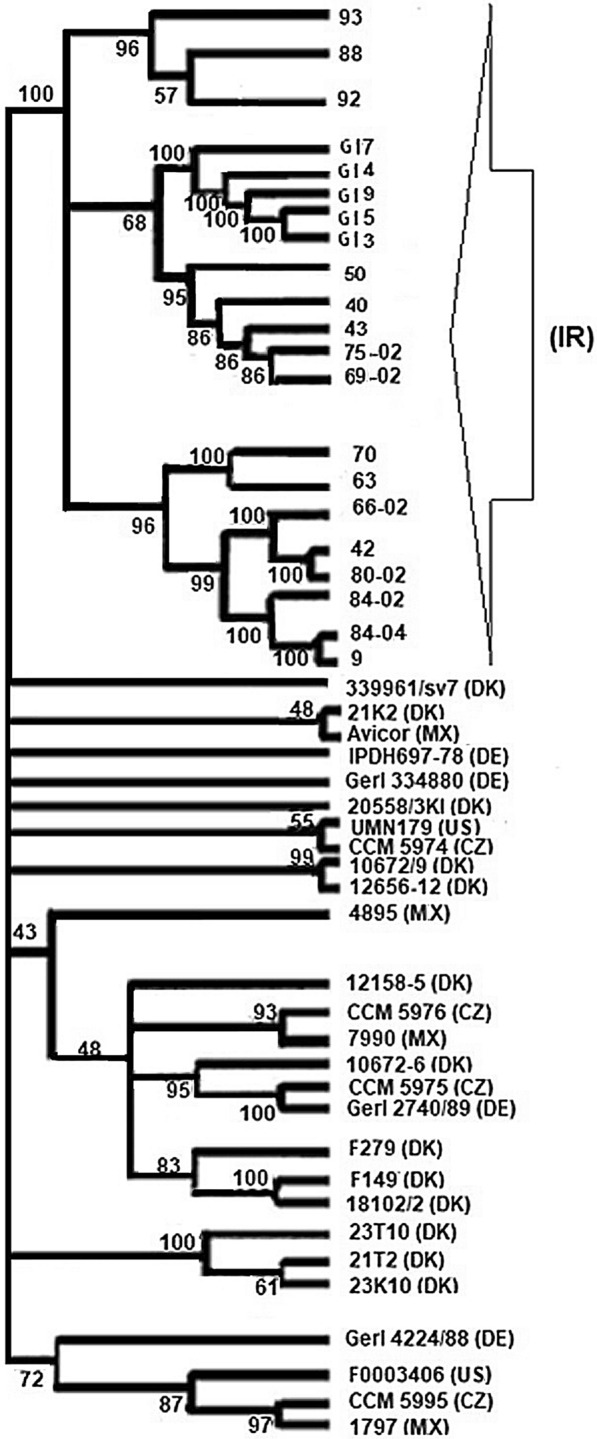
**Phylogenic tree of 48 strains of** ***G. anatis*** **based on the** ***gtxA*** **gene sequence comparisons.** The tree was composed of sequences from Iran (IR), Germany (DE), Denmark (DK), Czech Republic (CZ), USA (US) and Mexico (MX), which were compared using maximum likelihood. A total of 100 bootstrap replicates were included. Bootstrap values were indicated on the individual branches.

The *gtxA* gene sequence comparison revealed that all 21 genomes sequenced contained the *gtxA* gene with an overall sequence similarity among all of the strains from Iran, Denmark, Mexico, USA, Germany and Czech Republic of more than 92% at the DNA level. This indicates that *gtxA* has a highly conserved sequence across a broad range of epidemiologically unrelated strains. The results indicate that the phylogenic tree included separate groups whereof one branch included all the Iranian strains. Based on the phylogenetic analysis of the *gtxA* genes in all strains using maximum likelihood, the results indicate that the *gtxA* genes from the Iranian *G. anatis* strains were more closely related to one another (Figure 5).

### Antimicrobial susceptibility testing

The results of antimicrobial susceptibility testing of the 21 Iranian *G. anatis* strains against the eleven antibiotics listed in Table 3 indicate resistance to tetracycline (76.2%) and enrofloxacin (90.5%) to be very common. Among the 21 strains tested, 95.2% were susceptible to amikacin and chloramphenicol and 76.2% were susceptible to imipenem and gentamicin. The isolates were susceptible to doxycycline and trimethoprim/sulfamethoxazole with 81% and 90.5%, respectively. The strains were grouped as intermediate (71.4%) and susceptible (80.9) against first-generation and third-generation cephalosporins (cefazolin and ceftazidime), respectively. 66% of the tested strains were grouped as susceptible against amoxicillin/clavulanic acid (Table 3). Out of the 21 isolates investigated for tetracycline susceptibility testing, all except five strains (40, 43, 50, 69-02, 75-02) were resistant to tetracycline. All 16 tetracyclin resistant strains had the *tet*B gene in their genome (Figure 4). There were full agreement between the phenotypic and genotypic susceptibility testing results. Based on these results, the Iranian *G. anatis* isolates were considered multidrug resistant due to the resistance toward antimicrobials from at least three antimicrobial classes.

## Discussion

*Gallibacterium anatis* appears to be an under-diagnosed or ignored cause of infection in Iranian chickens although it may be a major disease related organism in the poultry industry. Isolation and identification of *Gallibacterium* from layer chickens exhibiting reproductive tract problems was reported for the first time in Iran in 2017 [35]. In the current study, *G. anatis* was isolated from chickens in Iran to investigate the presence and characteristics of the organisms, in order to provide useful information regarding the phylogenic relatedness, resistance to antimicrobials and presence of potential vaccine candidates in the Iranian *G. anatis* strains.

A total of 120 tracheal samples from chickens were subjected to culture-based and molecular identification methods, which resulted in identification of 84 strains of *G. anatis*. The flocks investigated represented all the different major production systems including battery-cage layers and broilers located in the leading poultry production provinces in Iran. As the vast majority of Iranian chickens are distributed from these regions to provinces with lower regional concentration of poultry, we considered it very likely that the widespread occurrence observed in the current investigation also applies to the remaining Iranian poultry production systems. Based on our results, the levels of biosecurity has an apparent influence on the occurrence of *G. anatis* infection in chickens as previously reported in Denmark, which indicated a negative correlation between biosecurity level and the presence of *Gallibacterium* [4].

We employed two different genotyping methods, PFGE and core genome SNP typing, to provide different information to elucidate the evolution and diversification of the *G. anatis* strains characterized.

A total of 71 *G. anatis* pulsotypes grouped into 24 clusters at an 87% similarity level. The results demonstrate a generally diverse PFGE pattern with no predominant pattern among the strains, which align well with previously published results where a considerable diversity was observed between isolates from different farms and even within individual animals [36]. On the contrary, in the present investigation, we also identified *G. anatis* identical PFGE profiles (clones) at different farms and on farms from different production systems, indicating a clonal spread or transmission among epidemiologically unrelated farms. To our knowledge this is the first report to reveal what appears to be clonal spread of *G. anatis* on a country-wide level.

The genetic diversity among the isolates within a farm might be due to insertions, deletions or point mutations in the ApaI restriction sites, which could lead to the observed variation in PFGE profiles. The finding is in line with results from typing of *E. coli* EHEC O157:H7 strains where distinct insertions or deletions in the XbaI restriction sites led to diversification in the PFGE profiles between the strains [37]. The chance of detecting more chromosomal mutations can be improved using more restriction enzymes leading to other fragment patterns, which provides a better estimate of genetic relatedness between strains [38, 39]. Additionally, accessory genes acquired by horizontal gene transfer can lead to rapid evolution and polymorphism among the isolated strains [40]. The chromosomal variability among the 71 Iranian isolates reveals a complex population of *G. anatis* representing a large genetic reservoir leaving the population able to adapt to diverse environmental conditions.

The detailed investigation on the genetic content of the Iranian *G. anatis* strains also provides valuable information in the prevalence study of the isolates investigated. Based on the core genome SNP differences between the 21 isolates, the strains grouped into seven clusters with similarity value > 97% indicates that they were highly conserved the core genome level and closely related to a common ancestor. Regarding the information on the spatio-temporal origin of the isolates, *G. anatis* strains from the Sari and Qom provinces were scattered throughout the SNP-based phylogenic tree whereas the Tehran isolates clustered at a 99.9% similarity level indicating a common ancestry. At inspection of the pulsotypes, all the isolates from the Tehran, Sari and Qom provinces were scattered throughout the dendrogram regardless of their geographical origin and production system. The comparison of the phylogenic trees generated by PFGE and SNP-based core genome indicates limited consistency between groupings obtained by PFGE and SNP-based core genome analysis. PFGE clustered relatively distantly related (based on core-SNP) strains together on the one hand while other seemingly closely related strains had relatively different pulsotypes. In example, the isolates from the Tehran province clustered closely together in the SNP-based core genome tree, while scattered all over the PFGE tree. This is likely caused by variations among the accessory genes acquired by horizontal gene transfer, which is a major driver of bacterial evolution and significant variation in genomic content. Based on the results obtained from these typing methods, the closely related strains that formed the clusters were scattered in different geographic locations which suggests that there is no significant correlation patterns between genetic relatedness and spatial distribution among the Iranian *G. anatis* isolates.

The WGS typing method also allowed identification of antibiotic resistance genes and assessment of the presence or absence of virulence factors, which may be useful at development of effective prophylactic measures against antibiotic resistant bacteria. Considering the previously proposed immunogens, the *gtxA*, *gab_1309* and *gab_2312* genes these were found in all the Iranian strains isolated suggesting that those traits are part of the core genome rather than within accessory genome. In example, the hemolytic property of the strains indicated that the *gtxA* gene was functionally expressed as the GtxA toxin antigen in all included strains, which supports its use as a vaccine candidate. The absence of *flfA* gene in eight strains may be due to the presence of a different fimbrial type or a truncated version of the gene as has been reported previously [16]. The absence or low prevalence of some virulence associated genes may be explained by their acquisition through horizontal gene transfer, which may have taken place in a subset of *G. anatis* strains only [41]. Due to the absence of *flfA* and *gab_2156* genes in some strains, they should not be considered as potential vaccine candidates against the Iranian *G. anatis*. A common presence of the *tetB* gene was demonstrated in the Iranian *G. anatis* strains and tetracycline resistance is widespread. Several studies support that the high level of antimicrobial resistance observed among bacterial isolates from chickens is related to the common use of antimicrobials for disease treatment and as growth promoters, often without prior veterinary consultation [42]. The *tetB* gene was absent in five isolates (50, 40, 43, 75-02, 69-02), which clustered together in the core SNP tree although originating from different sources supports the suggestion that specific traits can cause clustering of strains despite difference in origin [43]. The difference in gene content among the isolates suggests that they are under diversifying selection due to adaptation to host niches and interactions with the host immune system, although further analysis would be required to confirm this observation.

The antimicrobial testing results of the 21 *G. anatis* isolates, selected from broiler and battery-cage layer flocks indicated high frequency of resistance to tetracycline (76.2%) and enrofloxacin (90.5%), which was reported in previous studies [9, 44, 45]. Resistance to tetracycline reported for 92.0% of Danish *G. anatis* field strains [9]. Our result in testing sulfamethoxazole/trimethoprim (90.5% susceptible) was in agreement with studies reporting high sensitivity to the antimicrobial combination in 93% and 83% in *M. haemolytica* and *G. anatis* isolated from chickens, respectively [44, 45]. In agreement with our findings, a high susceptibility rate against gentamicin was reported for *G. anatis* strains in Germany [46]. Multidrug resistance in bacteria may be generated when multiple genes, each coding for resistance to a single drug, accumulate within a single cell. This accumulation occurs in the genome or on resistance (R) plasmids. Multidrug resistance may also occur by the increased expression of genes that code for multidrug efflux pumps, extruding a wide range of drugs. Based on the Resfinder results in this study, the presence of tetracycline resistance genes (*tetB*, *tetC*, *tetR*) was confirmed in the genome of the 16 strains sequenced, but the mechanisms of resistance to the other antimicrobials tested are currently unknown. Based on a previous study on *Gallibacterium*, horizontal gene transfer can be associated with the acquisition of resistance to antimicrobial agents [9]. Multidrug resistance may also occur by increased expression of genes like *ybhf-1* that code for multidrug efflux pumps to extrude different kinds of drugs [47]. Our investigation revealed resistance to antimicrobial agents in the Iranian *G. anatis* clades indicating there is a need for continued monitoring of the antimicrobial susceptibilities of *Gallibacterium* isolates in the Iranian poultry production systems. The common presence of the multidrug resistant strains of *G. anatis* should be taken into account as a potential risk factor in chicken production systems underlying an attempt to identify potential vaccine targets as long-term preventative solutions against *Gallibacterium*. Based on the distribution of the immunogens in all of the Iranian strains, GtxA, Gab_1309 and Gab_2312 appear to be the most promising vaccine candidates. We assessed the distribution and sequence variation of *gtxA* gene in the different strains of *G. anatis* from Iran and other parts of the world. The *gtxA* gene sequence alignments showed an overall similarity of 92% at the DNA level indicating that the gene is conserved amongst the isolates of diverse origin in time and space. Specifically for the Iranian strains the *gtxA* genes clustered in a group of highly similar sequences supporting vaccine development activities based on the GtxA toxin in Iran too.

In conclusion, using a phylogenomic approach we were able to type *G. anatis* strains from the predominant poultry production systems in Iran and show that multiple lineages of *G. anatis* exists. We also found that some clonal types seemed to be widely dispersed across different farms and production systems. The presence of multidrug resistant isolates and a high frequency of resistance genes including *tetB* clearly indicate that multidrug resistance is common among the Iranian *G. anatis* isolates, which warrants further investigations into alternative means to prevent and control the bacterium. Here the toxin gene *gtxA* appears to be a good candidate, which may be included into a new vaccine in attempt to develop prophylactic measures against this organism in Iranian poultry.

## Data Availability

The datasets supporting the conclusions of this article are included within the article.
